# Evaluating psychodynamic therapy for nail picking and biting: An approach to body focused repetitive behaviors

**DOI:** 10.1111/srt.70036

**Published:** 2024-08-27

**Authors:** Isabella J. Tan, Priya Agarwal, Stacy K. Nakell, Mohammad Jafferany

**Affiliations:** ^1^ Rutgers The State University of New Jersey New Brunswick New Jersey USA; ^2^ Robert Wood Johnson University Hospital New Brunswick New Jersey USA; ^3^ Lotus Therapy, LLC Austin Texas USA; ^4^ Central Michigan University/CMU Medical Education Partners Saginaw Michigan USA

Dear Editor,

Body‐focused repetitive behaviors (BFRBs) include conditions such as nail biting, skin picking (dermatillomania), and hair pulling (trichotillomania), which cause self‐inflicted physical harm, often to serve as a coping mechanism for stress, anxiety, or emotional discomfort.^1^, ^2^ BFRBs are estimated to affect 0.5% to 4.4% of the population; however, prevalence may be underreported as many patients do not seek treatment.^3^, ^4^ Risk factors include female gender, history of obsessive‐compulsive disorder, and trauma.^4^

BFRBs significantly impact skin health, leading to infections, scarring, and severe complications like septicemia and significant blood loss.^4^ Understanding the psychological roots of these behaviors is crucial for dermatologists, as addressing emotional conflicts can mitigate skin damage.

Herein, we present the case of a 29‐year‐old Caucasian woman who sought treatment for chronic onychophagia and onychotillomania which had persisted since adolescence. These behaviors were precipitated by her parents' divorce and her mother's abandonment. Recent stressors, including her father's death, exacerbated her condition, causing significant tissue damage (Figure 1). Diagnosed with excoriation disorder, her symptoms were linked to relational stress and unresolved grief, common in severe manifestations of these conditions.^1^, ^2^

**FIGURE 1:**
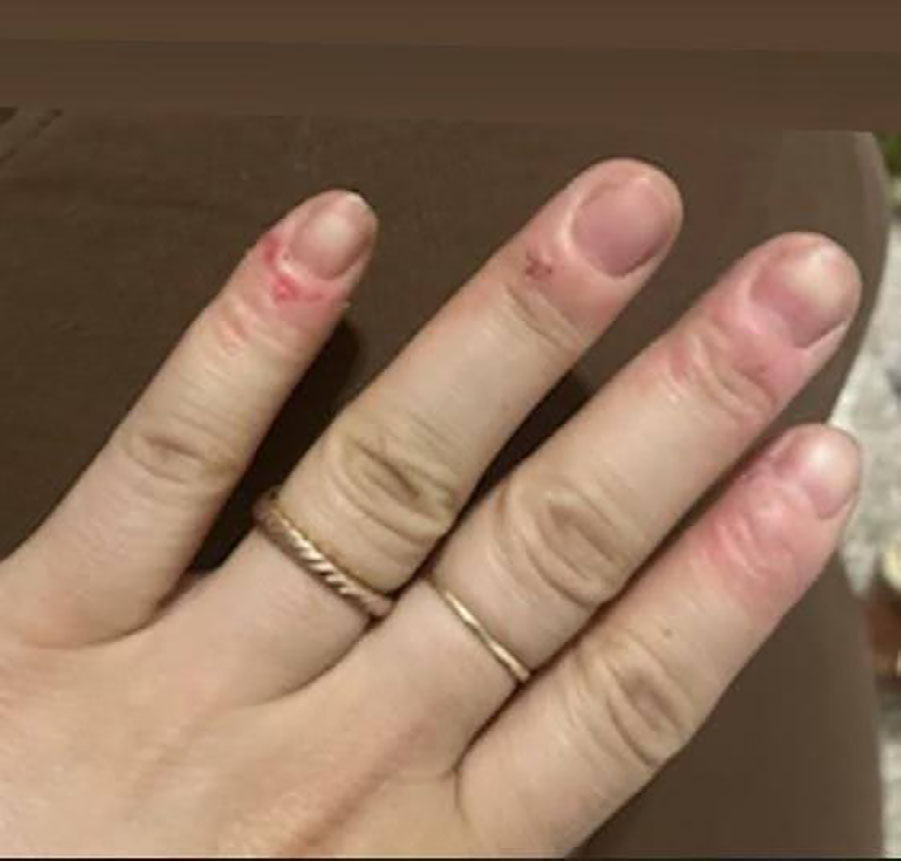
The patient's skin at the start of treatment.

The use of cognitive behavioral therapy (CBT), including habit reversal therapy and stimulus control, was considered for this patient. CBT; however, has historically focused on symptom reduction rather than addressing the underlying etiology of picking behaviors.^1^ Therefore, psychodynamic therapy, which has been shown to address both cognitive and behavioral manifestations, was utilized in this case.^1^ In this approach, the therapeutic relationship provided the foundation from which new emotional regulation skills could be internalized.

From this framework, treatment consisted of a safety phase followed by a focus on skill‐building and exploration. Initial sessions emphasized self‐compassion and understanding the function of nail picking as a coping mechanism. The patient explored past traumas, particularly related to her father's death and mother's absence. After 8 months of weekly therapy, the patient significantly reduced her nail‐picking and biting behaviors. She developed mindfulness, self‐compassion, assertiveness, and emotional modulation skills. Creative behavioral substitutions including nail care and nail art provided healthy alternatives. At a 4‐month follow‐up, she maintained progress and appreciated her newfound self‐awareness and resilience (Figure 2).

**FIGURE 2:**
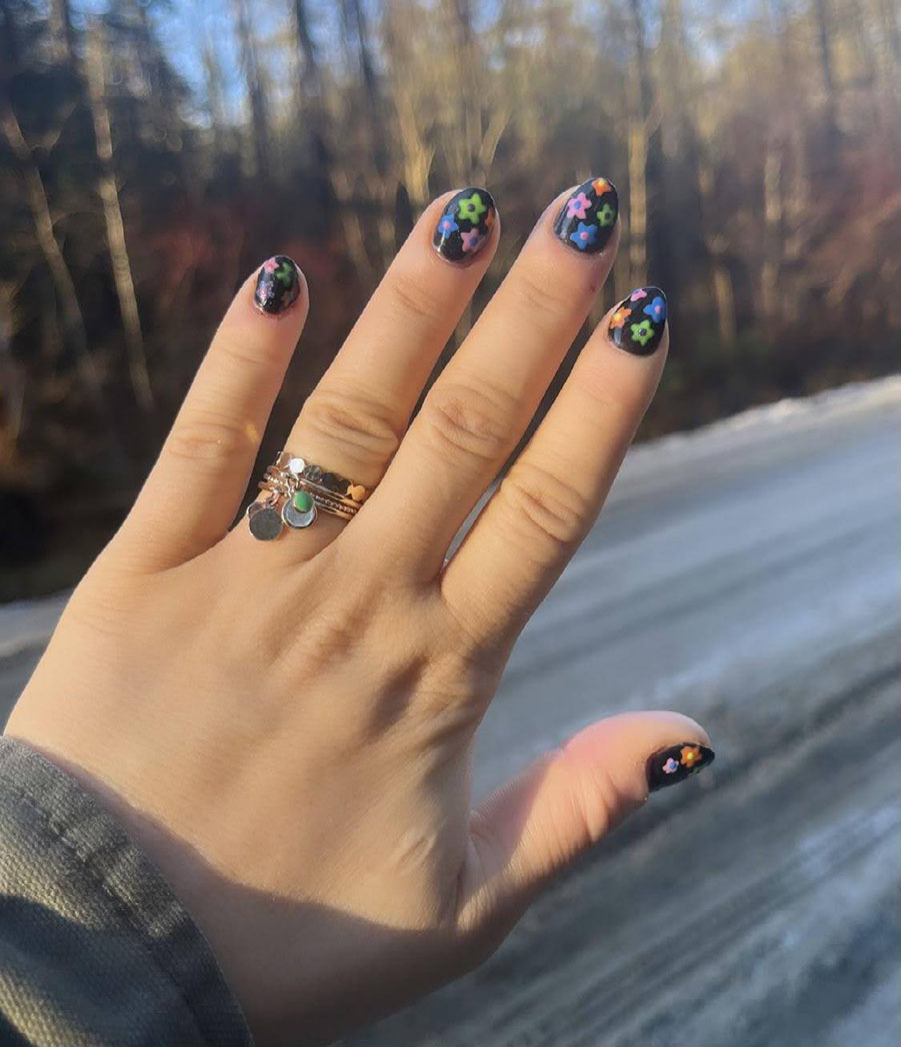
Four months into maintenance treatment. Creative expression through nail art promoted healing in this patient.

Notably, it has been found that use of a nail strengthener significantly increases nail structural elasticity, making them more flexible and less prone to breakage compared to untreated nails.^5^ Use of nail strengtheners, in conjunction with nail art therapy as described in our report can be beneficial in treating patients with onychophagia and onychotillomania, allowing patients to utilize grooming as a healthy outlet.

Psychodynamic therapy, including cognitive and behavioral tools, along with an emphasis on nail health, significantly reduced the patient's cuticle‐picking and biting behaviors by fostering self‐awareness, understanding defense mechanisms, and enhancing emotional regulation. This case underscores the importance of addressing emotional roots in psychodermatological issues and suggests that an integrative approach can lead to lasting symptom reduction in cases of severe BFRBs.

## CONFLICT OF INTEREST STATEMENT

The authors declare no conflicts of interest.

## CONSENT STATEMENT

Informed written consent was obtained from the patient. The patient's identity remains confidential throughout the report.

## Data Availability

This report is descriptive, based on clinical observations and narrative descriptions. As such, there are no data to be shared.
